# Which factors are associated with the functional recovery in patients undergoing endoprosthetic knee reconstruction following bone tumour resection? – A observational study

**DOI:** 10.1186/s40945-018-0052-1

**Published:** 2018-12-29

**Authors:** Mattia Morri, Debora Raffa, Daniela Vigna, Maria Barbieri, Elisabetta Mariani, Davide Maria Donati

**Affiliations:** IRCCS Istituto Ortopedico Rizzoli, Via Pupilli 1, 40136 Bologna, Italia

**Keywords:** Bone neoplasm, Knee endoprosthetic, Rehabilitation, Gait recovery, Postural balance

## Abstract

**Background:**

The aim of the present study was to explore whether control of balance and other factors were associated with functional recovery and walking performance in the short term in a group of patients receiving modular knee endoprosthetic reconstruction following bone tumour resection in order to provide effective suggestions for a new rehabilitation protocol.

**Methods:**

A cross-sectional study was carried out in the chemotherapy ward of an Italian hospital specialized in bone cancer. All patients consecutively treated using a modular knee endoprosthetic between January 2013 and February 2014 were included in the study. One year after surgery, various measuring instruments were used to assess the functional outcome achieved: Musculoskeletal Tumor Society rating scale, Toronto Extremity Salvage Score and specific motor tests of gait, such as gait speed and resistance. Data concerning the variables involved are as follows: bone resection, knee joint range of motion, quadriceps muscle strength and posture control. Statistical tests included correlation analysis (Pearson and Spearman correlation).

**Results:**

Balance control was significantly correlated to all the gait tests performed. Age, duration of chemotherapy and strength of the knee extensor muscles also showed a correlation. Conversely, joint range of motion and resection percentage did not show a significant correlation.

**Conclusions:**

Rehabilitation in patients undergoing knee joint reconstruction due to cancer should include balance control exercises, which involve not only the treated limb but address the entire sensory and motor system. This extends beyond the concept of treatment aimed at improving individual functions such as joint range of motion and muscular strength.

## Background

Knee reconstruction with modular endoprosthetic after resection of the distal femur or proximal tibia due to musculoskeletal tumour is becoming a more widely used lower limb salvage procedure by orthopaedic surgeons [1]. The increased 5-year survival rate, from 20 to 85% [2, 3], has improved surgical technique and the young age of patients affected by this disease have, over time, attracted growing interest in the functional outcome that can be achieved by this population of patients [2–5]. The success of surgery, regardless of the reconstruction method used, depends on removing the tumour mass with a wide margin, while at the same time, trying to preserve the best possible joint function [4, 6, 7]. The functional deficit resulting from surgery is due to two anatomical alterations: the removal of the joint unit along with the adjacent metadiaphysis and relevant muscles and, at the same time, the sacrifice of elements of the peripheral nervous system such as multiple sensory receptors, especially including mechanoreceptors. De Visser et al. [8] showed that the control of balance after surgery was reduced compared with that of a healthy population. Indeed, controlling an upright posture was more difficult due to lateral instability and asymmetry during weight bearing in gait. In the past, assessing the deficit of balance was not given due attention when evaluating its impact on functional recovery in these patients. Some studies performed on total knee arthroplasty in patients with arthritis showed how specific recovery of balance can also facilitate functional recovery [9, 10]. Physiotherapy, and especially kinesiotherapy, play a decisive part in guiding patient recovery, by minimizing the motor ability deficit and limited participation associated with disability [11]. In the recovery of articular excursion and muscle strength, Carty et al. (2009) [12] identified two priority objectives. The aim of the present study was to explore the association between balance and functional recovery in the short term in a group of patients receiving modular knee endoprostheses after bone tumour resection in order to provide effective suggestions for a new rehabilitation protocol. Other factors that might be important for postoperative recovery, such as age, quantity of bone resected, duration of postoperative chemotherapy, joint range of motion, and muscle strength, were also assessed.

## Methods

### Study design: Cross-sectional study

At a reference centre for bone tumour treatment, all patients consecutively treated using modular knee endoprostheses due to bone tumour located in the distal femur or proximal tibia between January 2013 and February 2014 were included in the study. One year after surgery, the patients underwent an assessment of the functional outcome obtained. Patients who suffered complications, such as local recurrence of the disease, prosthesis infection, mechanical failure of the prosthesis and problems concerning the administration of chemotherapy, were excluded from the study. The data listed below were collected by a hospital physiotherapist responsible for performing the assessment tests and filling in the measurement scales during the regular oncological and orthopaedic follow-up visits foreseen by our hospital’s outpatient clinics after surgery. The study was approved by the Ethics Committees of the Istituto Ortopedico Rizzoli n.0000393, 12/01/2018). The following variables were measured:

- bone resection, calculated as a percentage of resected bone in relation to the length of the femur or tibia according to the tumour localization;
- knee joint range of motion (ROM) [13], measured with the help of a manual goniometer;
- strength of the quadriceps muscle, measured using the Medical Research Council protocol [14] with a scale from 0, no muscle contraction, to 5, force against a maximum manual resistance.
- posture control, measured by a stabilometric platform (LorAn Engineering Srl – Sistema EPS-R1). The assessment test was performed by asking the patient to stand upright for 10 s on the platform with a distance between the medial edges of the feet of 10 cm and an angle of 10° between the sagittal plane and the line passing through the first toe. The platform recorded the speed of the centre of pressure (CoP) through the relationship between the total distance covered by the CoP and the duration of the sampled period. De la Torre et al. identified a good index of the activity required in this parameter to maintain stability. The test was repeated with eyes open (EO) and eyes closed (EC). To date, no uniformity is present in the literature on the methods used to carry out this testing, thus making it not possible to define a standard of evaluation [15–18].
- Various measuring instruments were used to assess the functional outcome achieved such as the specific level of disability and ability of gait:
- disability was assessed using the Musculoskeletal Tumor Society Rating Scale (MSTS) [19] for lower limb. This is a subjective non-parametric system that assesses several recovery aspects: pain, overall ability, emotional state, use of supports, gait ability and the way the patient walks. A score ranging from 0, for maximum disability, to 5, for maximum autonomy, is given; the minimum score is 0 and the maximum 30;
- the Toronto Extremity Salvage Score (TESS) [20] for lower extremity is a questionnaire self-administered by the patient and made up of 30 questions concerning patient motor ability when performing daily living activities, by recording the patient’s own impressions with regards to their ability. Each question is assigned a point ranging from a minimum of 1 to a maximum of 5. The overall score is expressed as a percentage;
- Specific gait tests, such as gait speed (expressed as time measured to walk a distance of 10 m, Graham JE 2008) [21], gait resistance (six minutes walking test, 6mWT) [22] and mobility (Time up and go, TUG) [23]. Beebe and collaborators (2009) [24] and Ginsberg et al. [25] showed the importance of using specific motor performance measures to highlight functional deficits that would otherwise remain underestimated.

### Statistical analysis

Statistical analysis was performed using the IBM SPSS Statistics v. 21. Statistical tests included correlation analysis (Pearson and Spearman correlation). Normality and linearity of data was assessed using scatterplots. Statistical significance was set at *P* < 0.05.

## Results

After one-year postsurgery, it was possible to contact 19 of the 22 patients treated with modular endoprosthesis. Four patients were excluded, of which three due to local recurrence and one due to fracture of the treated femur that required revision and replacement of the implant. Table 1 shows the sample characteristics, the variables taken into consideration and the results obtained from the study sample. Balance control was significantly correlated to all the gait tests performed. Age, duration of chemotherapy and strength of the knee extensor muscles also showed a correlation. Joint ROM and resection percentage demonstrate a strong positive association with respect to the MSTS score, but not a statistical significance. Correlations and outcomes are shown in Table 2.

**Table 1:** Patient characteristics, variables and functional results

Patient characteristics	
Age, years (SD)	27.5 (18)
Female, n. (%)	5 (33%)
Morphology, n. (%)	
Osteosarcoma	13 (87)
Condrosarcoma	2 (13)
Site of the tumor, n. (%)	
Femur	12 (80)
Tibia	3 (20)
Follow-up, months (SD)	12.9 (1.6)
% of bone resection, (SD)	37.8 (12.5)
Chemotherapy, months (SD)	7.4 (2.7)
Knee flexion, degree (SD)	104.7 (19.3)
Strengh of knee extensor, grade (0–5) (SD)	3.9 (0.9)
Postural Control – SCoP (mm/sec)	
Eyes open, mean (SD)	9.9 (3.6)
Eyes closed, mean (SD)	13.2 (5.7)
Functional results	
Disability index	
MSTS score (SD)	23.7 (4.3)
TESS (%)	86.3 (10.8)
Gait performance	
10-m test (sec)	8.7 (2.5)
6mWT (m)	421.6 (115.5)
TUG (sec)	8.9 (2.6)

*SCoP* speed of the centre of pressure

**Table 2:** Pearson Correlation between the functional results and patient specific factors

	Disability Index		Gait performance		
	MSTS	TESS	10-m test	6mWT	TUG
Age	−0,49	−0,69**	0,40	−0,51	0,53*
% of bone resection	−0,66	−0,08	0,35	−0,38	0,21
Chemotherapy, months	−0,09	−0,20	0,39	−0,60*	0,36
Knee flexion, degree	0,49	0,42	−0,25	0,39	−0,30
Strength of knee extensor, grade^a^	0,65**	0,27	−0,27	0,30	−0,29
Postural Control – SCoP (mm/sec)					
Eyes open, mean (SD)	−0,156	0,06	0,73**	− 0,69**	0,61*
Eyes closed, mean (SD)	−0,10	0,04	0,69**	− 0,64*	0,54

^a^Spearman correlation

*p value < 0,05

**p value < 0,01

## Discussion

The main aim of the study was to assess the role of balance control in functional recovery in patients treated using modular knee endoprostheses after bone tumour resection. Although the data collected do not allow an inferential statistical analysis with the necessary power, the relationships studied show some interesting findings from a rehabilitation point of view which should be further investigated by studies using larger samples.

De Visser et al. [8] had already highlighted the impaired ability in these patients to control a standing upright position compared with that of the healthy population, especially with eyes closed. The results of the present study highlighted a link between these parameters and functional recovery, specifically recovery and performance of gait (speed and resistance): increased difficulty in managing balance is associated with reduced gait performance (10 m-test, 6mwT, TUG). The statistical analyses showed correlations ranging from moderately to strongly significant (Table 2). The same correlation, however, was not found for the level of disability: MSTS score and TESS score do not appear to be associated with the speed of CoP.

Balance control is based on the integration of various sources of information, such as that from visual, vestibular and proprioceptive afferents. This information is necessary for the neuromotor system to be able to realize suitable movement schemes and respond to environmental stimuli. When a patient undergoes wide musculoskeletal resection, this system becomes disturbed and this deficit seems to influence recovery of the best gait performance. Currently, the recovery pathway and treatment methods in the postoperative period are not widely described in the literature and comparative data are limited. In clinical practice, the patient is mainly offered exercises aimed at the recovery of knee articulation and muscle strengthening. For patients with knee endoprosthesis, multiple biomechanical factors may be responsible for reduced stability. Exercises for two-leg standing that progressively become more challenging by modifying the surface or using increasingly unstable surfaces, closed-eye training or dual task exercises, such as throwing a ball and standing in an unstable position, can all be introduced during the rehabilitation process. Balance exercises, not only involving the treated joint but also the entire motor and sensory system, should be encouraged in these patients in order to achieve a better gait performance. Concerning patients undergoing total knee arthroplasty, De Liao et al. [9] and Piva et al. [10] showed how rehabilitation programmes that include specific balance exercises can be beneficial in terms of functional outcome (10-m, TUG). According to the data collected, other useful proposals can be advanced in order to plan postoperative rehabilitation.

Strength of the knee extensor muscles was significantly correlated to the MSTS score, whereas no correlation was found between muscle strength and specific gait tests. In Carty’s study [12], where restored strength was similar (mean 4.15; range 2–5), this correlation was significant both for MSTS and TESS. Benedetti et al. [26] had already highlighted a lack of correlation between strength of the knee extensor muscles and the biomechanical patterns that the patient was able to develop during gait, by showing that a strength deficit alone does not necessarily lead to a more impaired gait pattern.

In the present study, knee movement in flexion was correlated with MSTS and TESS, however, given the small sample tested, this does not provide a statistical significance. This finding differs from that of Carty et al. [12] who showed a correlation between joint ROM and TESS. In the present study, 87% of the patients had a ROM of > 90° and the mean flexion achieved was 104° (SD 19, range 75–150), which was in line with levels published by Tsuao (106°, SD 13) [4], but lower than the 120° (range 85–140) published by Carty [12]. This difference might also explain the different correlation found. At the same time, the lack of association between the range of motion of the knee and specific walking tests is possible since, for normal walking, a knee flexion of 60° is sufficient, which all patients were able to reach. For professionals who deal with the functional recovery of oncological-orthopaedic patients, rehabilitation should not be aimed just at improving a specific deficit, such as muscle strength or joint ROM, but also exercises based more on complete gait patterns where proprioceptive stimulus is a central element.

In the present analysis we also examined some elements that cannot be modified directly during the course of treatment, but should be borne in mind by the clinician for a better understanding of the progress of a patient’s recovery. Moreover, the duration of chemotherapy and patient age are factors that can influence gait resistance and a patient’s perception of autonomy, respectively.

Though this study presents some novel findings, there are limitations to consider, mainly, the study uses a small sample size, thus excludes the opportunity of a more in-depth statistical analysis. The rarity of this disease is one element that significantly influences the possibility to design research protocols on larger and more uniform samples to be able to perform multivariate statistical analyses. For this same reason, patients undergoing distal femur and proximal tibia resection were included in the observation group, even though their reconstruction features have different biomechanical and anatomical elements. Only 3 out of 15 patients were found who underwent proximal tibia resection. Finally, the evaluation of the postural control included the measurement of the speed of the CoP with a stabilometric platform. The authors’ choice to carry out a test lasting 10 s is a limitation of the study, even if in the literature the execution of this measurement is not uniform and it is not possible to define a standard mode of execution. Furthermore, the authors hypothesize that the average speed of CoP is partially affected by the duration of the test itself.

According to the present authors, however, the data examined still provide useful indications to guide physiotherapy treatment of these patients and may be able to guide the way for future research in this field.

## Conclusion

Rehabilitation for patients undergoing knee joint reconstruction due to cancer should include balance control exercises which involve not only the treated limb but address the entire sensory and motor system as well. This extends beyond the concept of treatment aimed at improving individual functions such as joint range of motion and muscular strength. To understand the actual benefit of this type of treatment and the best way to apply it, clinical trials are needed that take into account gait-specific motor abilities as a primary outcome.

## Abbreviation

6mWT: Six Minutes Walking Test
CoP: Centre of pressure
EC: Closed eyes
EO: Open eyes
MSTS: MusculoSkeletal Tumor Society
ROM: Range of motion
SCoP: Speed of the centre of pressure
SD: Standard Deviation
TESS: Toronto Extremity Salvage Score
TUG: Time Up and Go
